# Molecular evolutionary insight of structural zinc atom in yeast xylitol dehydrogenases and its application in bioethanol production by lignocellulosic biomass

**DOI:** 10.1038/s41598-023-29195-7

**Published:** 2023-02-02

**Authors:** Kentaroh Yoshiwara, Seiya Watanabe, Yasunori Watanabe

**Affiliations:** 1 Department of Bioscience, Graduate School of Agriculture, Ehime University, 3-5-7 Tarumi, Matsuyama, Ehime 790-8566 Japan; 2 Faculty of Agriculture, Ehime University, 3-5-7 Tarumi, Matsuyama, Ehime 790-8566 Japan; 3 Center for Marine Environmental Studies (CMES), Ehime University, 2-5 Bunkyo-Cho, Matsuyama, Ehime 790-8577 Japan; 4 Faculty of Science, Yamagata University, 1-4-12 Kojirakawa-Machi, Yamagata, Yamagata 990-8560 Japan

**Keywords:** Biochemistry, Biotechnology, Structural biology

## Abstract

Xylitol dehydrogenase (XDH) catalyzes the NAD^+^-dependent oxidization of xylitol into d-xylulose, and belongs to a zinc-dependent medium-chain dehydrogenase/reductase family. This protein family consists of enzymes with one or two zinc atoms per subunit, among which catalytic zinc is necessary for the activity. Among many XDHs from yeast and fungi, XDH from *Pichia stipitis* is one of the key enzymes for bioethanol production by lignocellulosic biomass, and possesses only a catalytic zinc atom. Despite its importance in bioindustry, a structural data of XDH has not yet been available, and little insight into the role of a second zinc atom in this protein family is known. We herein report the crystal structure of XDH from *P. stipitis* using a thermostabilized mutant. In the refined structure, a second zinc atom clearly coordinated with four artificially introduced cysteine ligands. Homologous mutations in XDH from *Saccharomyces cerevisiae* also stabilized and enhanced activity. The substitution of each of the four cysteine ligands with an aspartate in XDH from *Schizosaccharomyces pombe* contributed to the significantly better maintenance of activity and thermostability than their substitution with a serine, providing a novel hypothesis for how this zinc atom was eliminated.

## Introduction

The utilization of ethanol produced from plant biomass (so-called “bioethanol”), which is derived from the fixation of atmospheric CO_2_, as an industrial carbon source and car fuel is one of the most important research issues for the realization of a sustainable global environment. Bioethanol is mainly produced from agricultural crop biomass, the biological fermentation of which is easy, but it commercially competes as food and animal feed resources. Alternatively, “lignocellulosic biomass”, such as woods and agricultural residues, represents an attractive feedstock that consists of cellulose (60% of the mass), hemicellulose (30%), and lignin (10%). Hemicellulose comprises pentoses, such as d-xylose (and l-arabinose), as well as hexoses, and d-xylose accounts for approximately 25% of the total sugar content in lignocellulosic biomass^1,2^.

**Figure 1:**
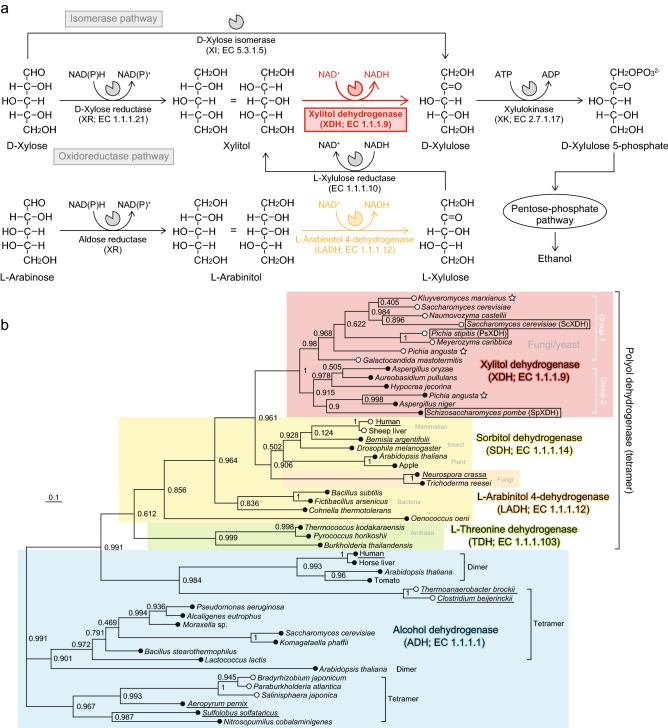
d-Xylose metabolism by yeast and fungi. (**a**) Metabolic network of d-xylose and l-arabinose. There are two routes of d-xylose metabolism, with XDH being involved in the oxidoreductase pathway. This pathway partially overlaps with l-arabinose metabolism, in which LADH belongs to the same protein family as XDH, described in (**b**). (**b**) A phylogenetic tree of the zinc-dependent MDR superfamily group. The number on each branch indicates the bootstrap value. Subfamilies of XDH, SDH, LADH, TDH, and ADH are colored in red, yellow, orange, yellow-green, and cyan, respectively. Open and closed circles at the end of each branch are enzymes in the absence and presence of structural zinc, respectively. XDHs with asterisks were enzymes from thermotolerant yeast. Proteins in the box were purified and characterized in the present study. Underlined proteins were used for discussions in the text.

Yeast, particularly *Saccharomyces cerevisiae*, has long been used to produce alcoholic beverages because of its ability to produce high concentrations of ethanol and its high inherent tolerance of ethanol. However, native strains cannot ferment d-xylose as a carbon source*.* Therefore, many studies have attempted to overcome the limitations associated with the utilization of d-xylose by introducing its metabolic pathway from other microorganisms^3,4^. Although the biological degradation of d-xylose in microorganisms is classified into phosphorylated and non-phosphorylated pathways^5,6^, only the former, which is further classified into two different pathways, is used for this purpose. In the “isomerase pathway”, d-xylose is directly converted into d-xylulose by d-xylose isomerase (XI; EC 5.3.1.5) without any cofactors (Fig. 1a). Although this pathway mostly operates in bacteria, a few fungi possess the bacterial type of XI^7^. Alternatively, in the “oxidoreductase pathway”, d-xylose reductase (XR; EC 1.1.1.21) catalyzes the reduction of the C1 carbonyl group of d-xylose, yielding xylitol as the product (Fig. 1a). Xylitol is then oxidized by xylitol dehydrogenase (XDH; EC 1.1.1.9) to give d-xylulose. Xylulokinase (XK; EC 2.7.1.17) commonly phosphorylates d-xylulose into d-xylulose 5-phosphate, which is metabolized further via the pentose-phosphate pathway. Although *S. cerevisiae* possesses the endogenous oxidoreductase pathway consisting of YHR104w (GRE3), YLR070c (XYL2), and YGR194c (XKS1; XK) as XR, XDH, and XK, respectively, the rate of D-xylose metabolism by strains overexpressing them has not yet reached industrially competitive levels^8^. Alternatively, XR and XDH genes from the native d-xylose-metabolizing yeast *Pichia stipites* (*Scherrsomyces stipites*; PsXR and PsXDH) are mostly used together with the endogenous XK gene.

XDHs from yeast and fungi, including PsXDH, belong to a polyol dehydrogenase (PDH) subfamily in the zinc-dependent group of the medium-chain dehydrogenase/reductase (MDR) superfamily^9,10^, together with sorbitol dehydrogenase (SDH; EC 1.1.1.14)^10–12^ and l-arabinitol 4-dehydrogenase (LADH; EC 1.1.1.12) from several organisms^13–15^ (Fig. 1b). Among them, LADH is involved in l-arabinose metabolism by fungi, which partially overlaps with the oxidoreductase pathway of d-xylose (Fig. 1a). All MDR enzymes utilize NAD^+^(H) or NADP^+^(H) as a cofactor, and one zinc atom with catalytic functions (so-called “catalytic zinc”) is present in the active center of PDH, alcohol dehydrogenase (ADH; EC 1.1.1.1)^16,17^, and l-threonine dehydrogenase (TDH; EC 1.1.1.103)^18^. Many PDHs and ADHs also have a second zinc atom (so-called “structural zinc”), which generally coordinates with four cysteine ligands. Since it is impossible to remove this zinc atom without the loss of stable folding or enzyme activity by site-directed mutagenesis^19,20^, “why” and “how” MDR enzymes appeared in the evolutionary stage in the absence of structural zinc remain unclear.

Although XR-XDH achieves higher metabolic fluxes than XI, the excretion of xylitol occurs during d-xylose fermentation by *S. cerevisiae*^21^. An intercellular redox imbalance due to the different coenzyme specificities of XR (with NADPH) and XDH (with NAD^+^) has been suggested as one of the main factors^4,5^. Furthermore, a relationship has been reported between stability and intercellular expression levels. Therefore, protein engineering to change (modify) coenzyme specificity and/or increase thermostability represents an attractive approach. A unique NADP^+^(H)-dependent SDH from insects^12^ is used as a reference enzyme, through which the complete reversal of coenzyme specificity towards NADP^+^ is achieved^22^. On the other hand, the introduction of four cysteine residues provided additional zinc-binding sites and significantly increased thermostability^22^.

Despite its importance in bioindustry, a crystallographic analysis of PsXDH has not yet been performed, and there is currently no structural evidence for protein engineering, particularly the artificial introduction of structural zinc. We herein report for the first time the crystal structure of PsXDH using a thermostabilized mutant. A second zinc atom coordinated with four artificially introduced cysteine ligands. The substitution of each of the four cysteine ligands with an aspartate in XDH from *Schizosaccharomyces pombe* contributed to the significantly better maintenance of activity and thermostability than their substitution with a serine, providing a novel hypothesis for how enzymes in the absence of structural zinc, such as PsXDH, appeared.

## Results

### Overall structure of the PsXDH_C4_ mutant

**Figure 2:**
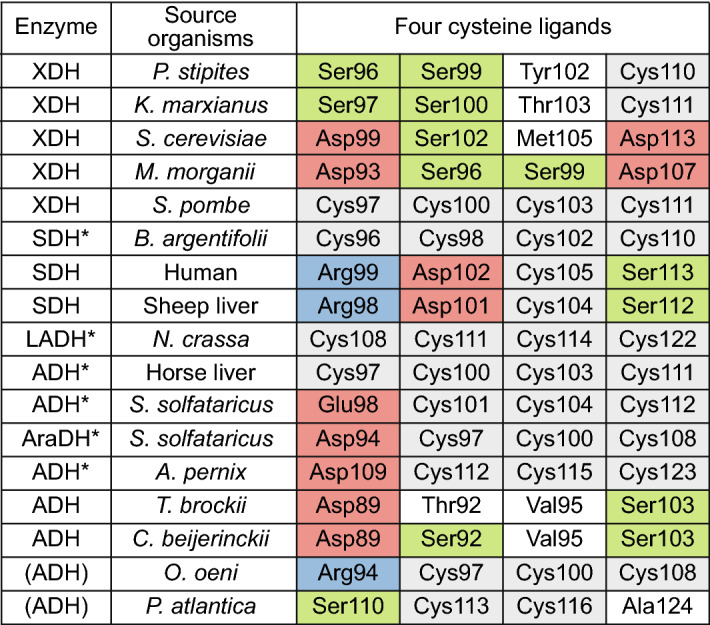
Four cysteine ligands for structural zinc binding in MDR enzymes. Cysteine, serine, acidic, and basic residues are shadowed in gray, yellow-green, red, and blue, respectively. Proteins with asterisks possess structural zinc based on a crystallographic analysis.

**Figure 3:**
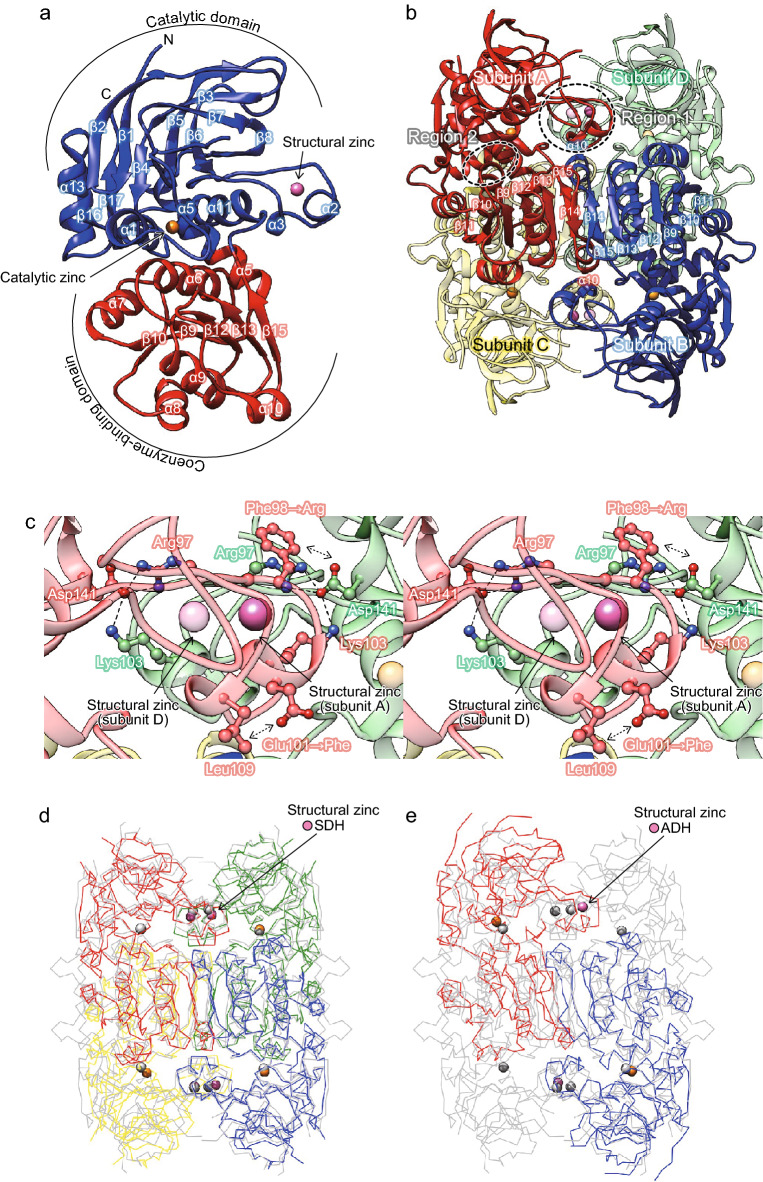
Crystal structure of the PsXDH_C4_ mutant. (**a**) Monomer structure. The catalytic domain housing both catalytic and structural zinc atom (orange and magenta spheres, respectively) is shown in blue, and the cofactor-binding domain is shown in red. The figure was prepared using PyMOL^49^. (**b**) Structure of the biological tetramer generated from crystallographic symmetry. The tetramer is a dimer of dimers (A/B and C/D). The dashed line ellipse indicates two contact regions 1 (**c**) and 2 between two dimers. In (**c**), hydrogen bonds are shown as black dashed lines. It is likely that the mutated Arg98 in subunit A can form a salt bridge with Asp141 in subunit D of another dimer, and the mutated Phe101 interacts with Leu109 within the same loop hydrophobically. Superimposed Cα traces of the structures of tetrameric PsXDH_C4_ (gray) on tetrameric SDH from silverleaf whitefly (1E3J) (**d**) and dimeric ADH from *Arabidopsis thaliana* (2CF5) (**e**).

The so-called PsXDH_C4_ mutant was previously constructed by substituting Ser96, Ser99, and Tyr102 with cysteine residues in the wild-type (WT) enzyme (Fig. 2)^22^. The crystal structure of the apo-form of PsXDH_C4_ was refined at 2.80 Å resolution after molecular replacement using the coordinates of SDH from sheep liver (PDB ID; 3QE3) as a research model^11^. Data collection and refinement statistics are summarized in Table 1. Each monomer contained a bidomain architecture composed of a large “catalytic domain” (Thr2-Val163 and Arg300-Pro362) and a smaller “coenzyme-binding domain” (Gly164-Phe299), with a large cleft separating them (Fig. 3a). The former consisted of an α/β fold with a similar structure to that in other MDR enzymes, and the latter had the characteristic α/β Rossmann fold. The region corresponding to Ser121–Gly124 within a long loop between α3 and β7 in the catalytic domain was not built owing to invisible density map.

**Table 1:** Data collection and refinement statistics.

	XDH C4 apo form (PDB code 7Y9P)
Data collection	
Space group	*P*622
* a,b,c* (Å)	158.27, 158.27, 65.50
Wavelength (Å)	1.28000
Resolution range (Å)	50–2.80 (2.95–2.80)
* R*merge	0.457 (4.19)
* R*meas	0.461 (4.23)
CC1/2	0.997 (0.856)
* I/*σ (*I*)	16.6 (2.6)
Completeness (%)	100 (100)
Redundancy	57.24 (60.46)
Refinement	
* R*/*R*free	0.223/0.274 (0.343/0.367)
No. of atoms	
Protein	2618
Zinc	2
Glycerol	6
Sulfate ion	5
Polyethylene glycol	7
Water	15
*B*-factors (Å2)	
Protein	64.08
Zinc	87.95
Glycerol	63.15
Sulfate ion	78.36
Polyethylene glycol	66.54
Water	50.91
r.m.s. deviations	
Bond lengths (Å)/angles (º)	0.004/0.688
Ramachandran plot	
Favored/allowed/outliers (%)	97.17/2.83/0.00
Rotamer outliers (%)	2
Clashscore	8

Analytical size-exclusion chromatographic studies demonstrated that PsXDH_C4_ formed a homotetramer in solution. The four subunits (A–D) in the biological tetramer generated from crystallographic 222 symmetry were regarded as a dimer of identical A/B and C/D dimers (Fig. 3b). The contact region between each monomer of the dimer was strand β14, which formed a seven parallel β-sheet (β11–β10–β9–β12–β13–β15–β14), and packed antiparallel with strand β14 of the second monomer. During the formation of the tetramer, the subunits A/B dimer contacted the subunits C/D dimer at two regions. Region 1 corresponded to a loop that protruded from the catalytic domain, at which a zinc atom other than the catalytic zinc atom bound, as described below (Fig. 3c). The side chain of Arg97 in subunit A (D) formed a salt bridge with the side chain of Asp141 in the same subunit A (D), which also interacted with the side chain Lys103 in subunit D (A) of the other dimer; symmetrical interactions are shown in parentheses. Region 2 was located between two loops of the coenzyme-binding domain in subunit A (C), and one helix of the catalytic domain in subunit C (A). Therefore, these two types of contact were achieved by different subunits in the opposing dimers.

**Figure 4:**
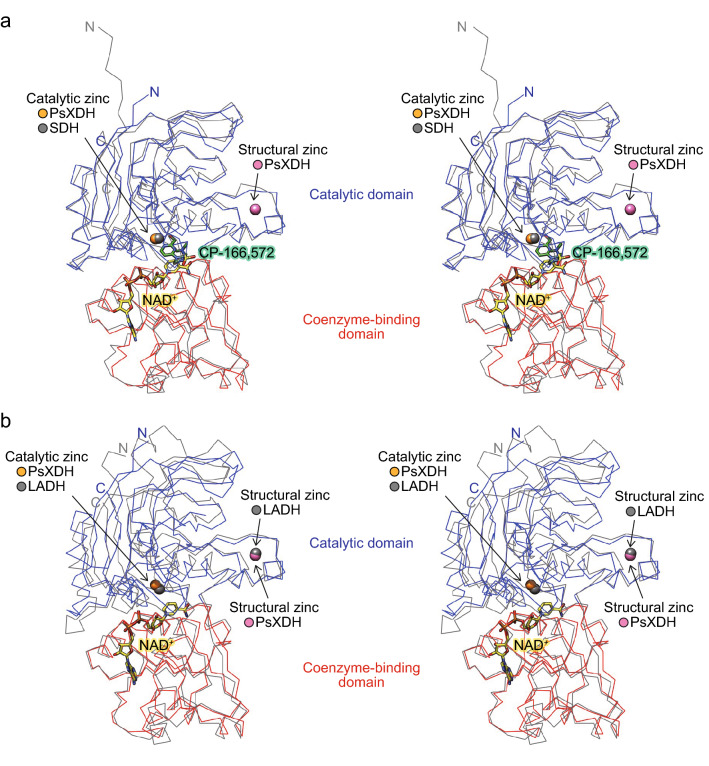
Stereo view of the superimposed Cα traces of the coenzyme binding domains of PsXDH_C4_ and SDH from human in complex with the competitive inhibitor (CP-16657213) and NAD^+^ (1PL6; gray) (**a**), or LADH from *Neurospora crassa* in complex with NAD^+^ (3M6I; gray) (**b**).

As described in “Introduction”, the zinc-dependent MDR superfamily group is phylogenetically classified into three subclasses; PDH, ADH, and TDH (Fig. 1b). As expected, higher (structural-based) sequence homologies were noted in the same PDHs (r.m.s.d. values of 1.3–2.2 Å and sequence identities of 36–42%) than in ADHs and TDHs (r.m.s.d. values of 2.1–3.5 Å and sequence identities of 21–31%) (Fig. 4 and Supplementary Table S1). PDHs and TDHs, and ADHs from bacteria and archaea are homotetramer with the same crystallographic symmetry as PsXDH_C4_ (Fig. 3d). On the other hand, ADHs from eukaryote and plant tend to be homodimer with two subunits (Fig. 1b), which corresponds to the A/B (or C/D) dimer of tetrameric MDR enzymes; therefore, neither regions 1 nor 2 contribute to formation of the tertiary structure (Fig. 3e).

### Catalytic zinc binding in the PsXDH_C4_ mutant

**Figure 5:**
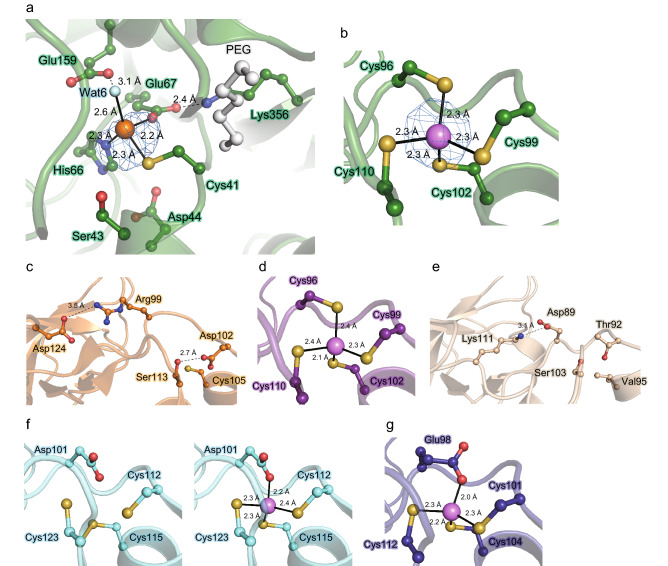
Zinc binding in the PsXDH_C4_ mutant. (**a**) Amino acid residues involved in chelating the catalytic zinc atom in the active site, together with some neighboring residues. PEG is derived from crystallization solution. Anomalous difference Fourier maps, contoured at 3.0 σ and 10 σ, suggest peaks for the catalytic zinc (**a**) and structural zinc atoms (**b**), respectively, and are shown as a blue mesh. (**b**) Structural zinc binding sites, and comparisons with SDH from human (1PL6) (**c**), SDH from silverleaf whitefly (1E3J) (**d**), and ADHs from *T. brockii* (1YKF) (**e**), *A. pernix* (1H2B) (**f**), and *S. solfataricus* (1JVB) (**g**). In (**f**), there are two structures in the presence of zinc (right panel) and in its absence showing the disulfide bond (left panel).

An anomalous difference Fourier map using data collected at the K-edge of zinc (wavelength of 1.280 Å) showed two clear peaks within the electron density map of PsXDH_C4_ (Fig. 5a,b). Among them, one peak, located at the bottom of the catalytic domain, corresponded to catalytic zinc, based on inference from other related structures (Fig. 4). Catalytic zinc was coordinated by interactions with Cys41 (distance of 2.3 Å), His66 (2.3 Å), Glu67 (2.2 Å), and a water molecule (Wat6; 2.6 Å) (Fig. 5a). The carboxyl group of Glu67 further formed a hydrogen bond with the side chain of Lys356, and nearby Glu159 was linked to the zinc atom through the wat6 molecule. All of these residues (and neighboring Ser43 and Asp44) were highly conserved in PDH members. XDH from the yeast *Galactocandida mastotermitis* (54% sequence homology with PsXDH) contained ~ 6 Mg^2+^ ions, which were selectively removed by dialysis without a loss of activity^23^. No magnesium ions were found in the crystal structure of the apo-form of PsXDH_C4_.

### Second zinc binding in the PsXDH_C4_ mutant

As described above, PsXDH_C4_ contained another peak in the anomalous difference Fourier map at the K-edge of zinc (Fig. 5b). Since binding sites were artificially constructed, another metal may have (concomitantly) been present; cobalt may have been alternatively introduced into the same binding sites of catalytic zinc as the native enzyme of ADH^16^. On the other hand, in previous biochemical study, the zinc content of the PsXDH_C4_ mutant was estimated to be ~ 1.9 mol of zinc/mol of subunit, which was close to 2.0, by atomic absorption spectrophotometry^22^. Furthermore, since zinc compounds were not used in the purification or crystallization protocols, we concluded that the metal must be zinc and intrinsically contained in the protein. To the best of our knowledge, this is the first structural evidence for artificially introducing structural zinc into MDR superfamily enzymes.

The second zinc atom was bound within a loop that protruded from the catalytic domain of PsXDH_C4_ (Fig. 3a), at which it was ligated by the enzyme residues Cys96, Cys99, Cys102, and Cys110 (distance of 2.3 Å) (Fig. 5b). Superimposition to the crystal structures of other MDR enzymes revealed that this zinc atom was equivalent to (inherently bound) structural zinc (Fig. 4b), and there was no significant difference in their binding loops regardless of zinc (Fig. 5c–g). SDH from humans and ADH from *Thermoanaerobacter brockii* possessed a salt bridge and/or hydrogen bond inside and/or outside of this loop (Fig. 5c,e).

**Figure 6:**
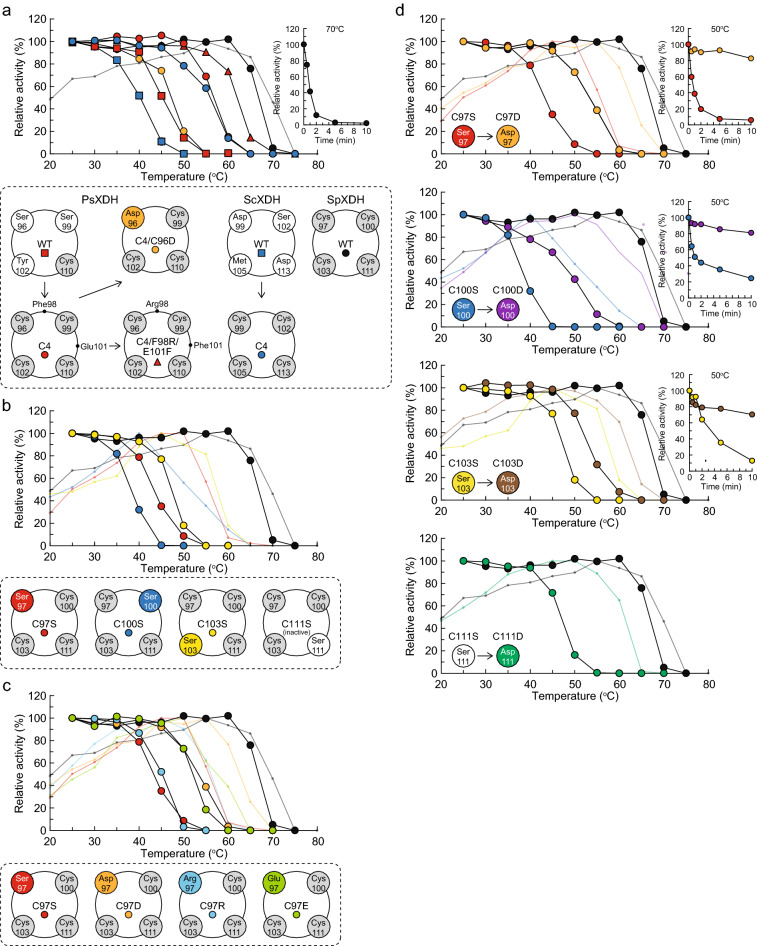
Courses of thermal inactivation. Purified enzymes were dialyzed against 50 mM Tris–HCl (pH 8.0) at 4 °C overnight. Dialyzed enzymes were incubated for 10 min at each temperature or at the indicated temperature for each time (inset). Enzyme activities were shown as relative average values (*n* = 3) expressed as a percent of the controls without a heat treatment. The background graph shows the effects of temperature on activity. (**a**) WT and mutants of PsXDH and ScXDH and WT of SpXDH. (**b**) Four serine mutants of SpXDH. (**c**) Four Cys97 mutants. (**d**) Four aspartate mutants and comparisons with each serine mutant.

When Phe98 and Glu101, neighboring residues of four cysteine ligands, were both substituted to arginine and phenylalanine residues in the PsXDH_C4_ mutant, respectively, the resultant double mutant (C4/F98R/E101F) further increased thermostability (Fig. 6a)^24^. Mutated Arg98 in subunit A may have formed a salt bridge with Asp141 in subunit D of the other dimer, and mutated Phe101 hydrophobically interacted with Leu109 within the same loop to significantly enhance dimer-dimer interaction(s) for the formation of tetramers (double-headed dashed arrow) (Fig. 3c).

### Introduction of four cysteine ligands for structural zinc in ScXDH

As described in “Introduction”, the YLR070c protein from *S. cerevisiae* (45% sequence homology with PsXDH; Fig. 1b) functions as XDH in the endogenous d-xylose pathway^8,25^, and possessed none of the four cysteine ligands (Fig. 2); therefore, we designed it the D99C/S102C/M105C/D113C mutant (referred to as ScXDH_C4_). Each WT and C4 mutant enzyme of ScXDH (and also PsXDH) was successfully expressed in *Escherichia coli* cells and purified using the same procedure as that for PsXDH. The *k*_cat_/*K*_m_ values of ScXDH_C4_ and PsXDH_C4_ for xylitol were 7.3- and 4.3-fold higher, respectively, than those of ScXDH_WT_ and PsXDH_WT_, and these differences were attributed to 10- and 22-fold higher *k*_cat_ values, respectively (Table 2). The inactivation of ScXDH_C4_ or PsXDH_C4_ was not detected after an incubation at 45 °C for 10 min, whereas the activities of each WT enzyme were decreased to 89 and 48%, respectively, by the same treatment (Fig. 6a). Collectively, the introduction of four cysteine ligands increased activity and thermostability in not only PsXDH, but also ScXDH, which appeared to be due to the introduction of a second zinc atom.

**Table 2:** Kinetic parameters of WT and mutant XDH enzymes for xylitol.

Enzyme	Mutation(s)	Pattern^a^	*Km* (mM)	*kcat* (min^−1^)	*kcat/Km* (min^−1^ mM^−1^)	Relative value (%)^e^	*Tm* value (°C)^f^
PsXDH	WT	S–S–Y–C	2.05 ± 0.20^b^	37.8 ± 3.5	18.4 ± 0.2	100	45.2 (0.0)^g^
	S96C/S99C/Y102C	C–C–C–C	4.94 ± 1.62^b^	384 ± 111	79.1 ± 4.0	430	56.8 (+ 11.6)
	S96C/S99C/Y102C	C–C–C–C	4.94 ± 1.62^b^	384 ± 111	79.1 ± 4.0	430	56.8 (+ 11.6)
	S96D/S99C/Y102C	D–C–C–C	11.0 ± 1.2^c^	550 ± 38	50.2 ± 1.7	270	47.2 (+ 2.0)
ScXDH	WT	D–S–N–D	1.82 ± 0.42^b^	1.09 ± 0.19	0.605 ± 0.042	100	40.2 (0.0)
	D99C/S102C/M105C/D113C	C–C–C–C	5.44 ± 0.78^b^	24.1 ± 3.0	4.44 ± 0.10	730	55.9 (+ 15.7)
SpXDH	WT	C–C–C–C	6.10 ± 0.34^c^	417 ± 18	68.5 ± 1.0	100	66.8 (0.0)
	C97S	S–C–C–C	12.3 ± 2.3^c^	362 ± 45	29.6 ± 1.6	43	43.3 (− 23.5)
	C100S	C–S–C–C	16.2 ± 5.0^c^	12.1 ± 4.0	0.743 ± 0.030	1.1	38.2 (− 28.6)
	C103S	C–C–S–C	9.01 ± 0.74	125 ± 6	14.0 ± 0.4	20	47.3 (− 19.5)
	C111S	C–C–C–S	N.D.^d^	N.D	N.D	N.D	N.D
	C97R	R–C–C–C	8.24 ± 1.42^c^	50.8 ± 5.7	6.21 ± 0.35	9.1	45.2 (− 21.6)
	C97E	E–C–C–C	13.4 ± 0.8^c^	3.26 ± 0.17	0.242 ± 0.003	0.4	52.1 (− 14.7)
	C97D	D–C–C–C	10.4 ± 0.1^c^	407 ± 3	39.0 ± 0.4	57	53.4 (− 13.4)
	C100D	C–D–C–C	8.26 ± 0.89^c^	130 ± 9	15.8 ± 0.6	23	48.5 (− 18.3)
	C103D	C–C–D–C	10.5 ± 0.7^c^	469 ± 18	44.6 ± 1.4	65	53.0 (− 13.8)
	C111D	C–C–C–D	9.76 ± 0.15^c^	216 ± 4	22.1 ± 0.1	32	47.0 (− 19.8)

Activity was assayed spectrophotometrically. Values are means ± SD, *n* = 3.

^a^Four amino acid residues at equivalent positions to Ser96, Ser99, Y102, and Cys110 in PsXDH.

^b,c^Eight different concentrations of xylitol between 0.1 and 1 mM (b) or 1 and 10 mM (c) in the presence of 1.5 mM NAD + were used.

^d^Not assessed due to trace activity.

^e^Relative value of *k*cat/*K*m to each WT.

^f^The apparent half denaturation temperature obtained from Fig. 5

^g^The difference from each WT enzyme.

### Serine mutants of four cysteine ligands for structural zinc in SpXDH

The hypothetical protein SPBC1773.05c from *S. pombe* (40% sequence homology with PsXDH; Fig. 1b) had four cysteine ligands at positions 97, 100, 103, and 111 (Fig. 2); therefore, we selected it as a target for the enzyme in the presence of structural zinc. The *k*_cat_/*K*_m_ value of xylitol was similar to that of PsXDH (31.3 and 79.9 min^−1^ mM^−1^, respectively), suggesting its function as XDH (referred to as SpXDH) (Table 2). To elucidate the physiological role of structural zinc, we initially substituted each cysteine ligand with a serine residue. A gel filtration analysis using the sample purified by Ni–NTA revealed that, compared with the WT, the ratio of active molecular species with tetramer structure was significantly decreased. Therefore, we performed a kinetic analysis using the fraction with the highest activity (Supplementary Fig. S1).

Among the four serine mutants, the *k*_cat_/*K*_m_ values of the C97S and C103S mutants (29.6 and 14.0 min^−1^ mM^−1^) were similar to that of the WT enzyme (68.5 min^−1^ mM^−1^), whereas that of the C100S mutant was markedly lower (0.743 min^−1^ mM^−1^) and the C111S mutant was completely inactive (Table 2). A heat treatment analysis indicated their significant decrease of thermostability; the half-live time for inactivation at 50 °C was estimated to be within 1 min (Fig. 6b,d), whereas the inactivation of WT was not detected by the same treatment. Similar results were observed at the optimum temperatures for activity. Collectively, these results indicated that all serine mutations markedly affected thermostability, and that the four cysteine ligands had varying impact on the levels of activity.

### Other mutants of four cysteine ligands for structural zinc in SpXDH

In some (putative) ADH subfamily enzymes in the MDR superfamily, one of the four cysteine ligands was substituted with an aspartate, glutamate, or arginine residue; D-C-C-C, E-C-C-C, or R-C-C-C, respectively (Fig. 2). In the crystal structures of (hyper)thermophilic archaeal enzymes, aspartate and glutamate residues coordinated with structural zinc (Fig. 5f,g)^26–29^. Therefore, Cys97 in SpXDH was changed to design the C97D, C97E, and C97R mutants. Among them, the courses of the thermal inactivation of the C97D and C97E mutants were enhanced (Fig. 6c).

An aspartate residue was frequently detected in some substitution patterns of the four cysteine ligands in MDR enzymes; D-S-M-D, D-S-S-D, and R-D-C-S. (Fig. 2). Therefore, Cys100, Cys103, and Cys111 in SpXDH were further substituted with an aspartate. The *k*_cat_/*K*_m_ values of the C97D, C100D, and C103D mutants increased and were 57%, 23%, and 65% that of WT, respectively (Table 2). Furthermore, the C111D mutant was significantly active, which differed from the C111S mutant. In the heat treatment analysis at 50 °C, losses in activity of 27%, 50%, and 23% were observed in the C97D, C100D and C103D mutants, respectively, which were less than those in each serine mutant (91%, 100%, and 82%), and their half-live times for inactivation at 50 °C were estimated to be longer than 1 h (Fig. 6d). Similar results were obtained for the optimum temperatures for activity. Regarding the C97D and C103D mutants, samples purified by Ni–NTA contained a large amount of the active tetramer, which may have been due to thermostabilization, as described below (Supplementary Fig. S1).

To investigate the effects of the aspartate ligand in more detail, (the mutated) Cys96 in PsXDH_C4_ was further changed to an aspartate residue. The resultant C4/S96D mutant (equivalent to the S96D/S99C/Y102C mutant) exhibited similar thermotolerance to WT (Fig. 6a), whereas the *k*_cat_/*K*_m_ value increased by 2.7-fold, which was caused by a marked increase in the *k*_cat_ value, similar to the C4 mutant (Table 2). On the other words, a change from C4 to C4/S96D in PsXDH had similar effects on WT and the C97D mutant of SpXDH, suggesting no difference of functions between artificial and inherent structural zinc.

### Intracellular expression level of XDH

**Figure 7:**
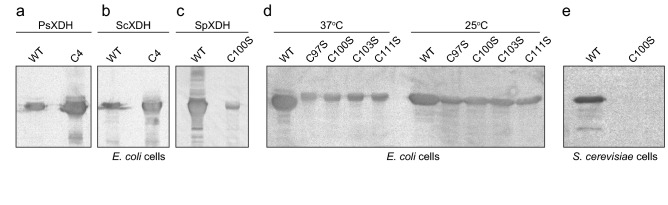
Intercellular expression level by an immunoblot analysis. Fifty micrograms each of cell-free extracts of transformed *E. coli* (**a**–**d**) or *S. cerevisiae* cells (**e**) was applied. A Western blot analysis was performed using the ECL Western blotting system (GE Healthcare) and Anti-Penta-His antibody (Qiagen) according to the manufacturer’s instructions.

A Western blot analysis using an anti (His)_6_-tag antibody showed that the PsXDH_C4_, ScXDH_C4_, and SpXDH_WT_ proteins were more highly expressed in *E. coli* cells at 37 °C than the PsXDH_WT_, ScXDH_WT_, and SpXDH_C100S_ proteins, respectively (Fig. 7a–c). When any of the four cysteine ligands in SpXDH was substituted with a serine residue, translational levels in *E. coli* cells at 25 °C were higher than those at 37 °C, whereas no significant difference was observed between them (Fig. 7d). Therefore, the peak with a high molecular weight in gel filtration using the sample purified by Ni–NTA appeared to be due to the aggregation of XDH (but not contaminant proteins) (Supplementary Fig. S1). Collectively, these results suggest a relationship between stability in vitro and intercellular expression levels in vivo.

## Discussion

### Molecular evolution of structural zinc

Only two studies previously investigated the artificial removal of structural zinc by site-directed mutagenesis, similar to the present study. Mutations in any of the four cysteine ligands to an alanine residue in ββ and χχ ADHs from humans^19^ or phenylacetaldehyde reductase (long-chain ADH) from *Corynebacterium* sp.^20^ resulted in no expression in *E. coli* cells or a marked decrease in activity to less than 4% that of the WT enzyme, suggesting the impossibility of removing the zinc atom without the loss of stable folding or enzyme activity. Alternatively, the C97S and C103S mutants of SpXDH maintained folding and activity; however, their thermostabilities decreased. In other words, if decreased stability is not a significant issue for enzyme function under physiological conditions, such a mutation may be neutral (but not negative). In spite of the (possible) removal of structural zinc, all SpXDH mutants maintained similar thermostabilities to PsXDH_WT_ and ScXDH_WT_ (Fig. 6a,b).

Any substitution(s) of the four cysteine ligands with an aspartate (and glutamate) residue in SpXDH_WT_ prevented a decrease in thermostability (the C97D, C100D, and C103D mutants), and/or enhanced correct structural folding (the C111D mutant). These acidic residues may have been alternatively coordinated to structural zinc, similar to some MDR enzymes (Fig. 5f,g)^26–29^. In other words, the four cysteine ligands may have been primarily modified via an aspartate residue, but not by random mutations in any residue(s). Since the structural lobe surrounding zinc formed one of the major points of contact in the XDH tetramer (Fig. 3b,c), a serine residue with a similar sized side chain to cysteine must have contributed to the maintenance of the integrity of this lobe after the loss of zinc. Therefore, the aspartate and serine residues, which are often found in the substitution patterns of the four cysteine ligands in PDHs, may be traced for the hypothetical evolutionary process; S-S-Y-C, S-S-T-C, D-S-M-D, D-S-S-D, and R-D-C-S (Fig. 2).

The introduction of the four cysteine ligands increased thermostability not only in PsXDH (S-S-Y-C), but also in ScXDH (D-S-M-D) (Fig. 6a). Since C4 mutations reversely mimic the molecular evolution described above, and do not have to be generated by a random mutagenesis method, this strategy may be broadly applicable to other MDR enzymes. XDHs from yeast and fungi are further classified into two groups, which correspond to enzymes in the absence (group 1) or presence of structural zinc (group 2), respectively (Fig. 1b). Among them, group 1 contains some enzymes from “thermotolerant” yeasts with the ability to grow and ferment at higher temperatures (50 °C), including *Kluyveromyces marxianus*^30^ and *P. angusta*^31^. Their thermostabilities are similar to PsXDH_C4_, indicating that these properties were acquired later by a strategy other than C4 mutations, such as refining of the structural zinc binding loop^24^, as described above (Fig. 3c).

### Application of bioethanol production by lignocellulose biomass

Although *S. cerevisiae* co-expressing the PsXR and PsXDH genes ferments (metabolizes) d-xylose, additional genetic introductions and/or deletions have been shown to result in an increased ethanol yield, concomitant with a decreased byproduct yield, including xylitol, glycerol, and acetate^32–34^. Another strategy is to modify the intercellular amount of XDH (and also XR) by plasmid copy numbers and the promoter control^35,36^. Alternatively, since increases in genetic expression levels may be (partially) compensated for by the intercellular lifetime of the translated protein (Fig. 7a–d), SpXDH may be significantly useful for d-xylose fermentation because of its markedly higher thermostability than not only PsXDH_WT_, but also PsXDH_C4_; the optimum temperature for activity was 55–65 °C and thermal inactivation was eventually observed at 70 °C (Fig. 6a). In a preliminary experiment, the SpXDH_WT_ gene was successfully expressed in *S. cerevisiae* cells under a constitutive phosphoglycarate kinase (PGK) promoter^37^, whereas no expression of the thermolabile C100S mutant was noted (Fig. 7e).

l-Arabinose accounts for approximately 28% of the hemicellulose fraction of corn fiber (14%). The efficient fermentation of l-arabinose by *S. cerevisiae* has been achieved by using the bacterial pathway consisting of AraABD^38^. On the other hand, the co-expression of LADH and l-xylulose reductase genes, involved in the fungal pathway (Fig. 1a), along with PsXR, PsXDH, and ScXK enabled *S. cerevisiae* to ferment l-arabinose; however, ethanol production occurred at a very low rate^39^. PsXDH exhibited native activity for l-arabinitol (data not shown), and showed high sequence homology with XDH from *Meyerozyma caribbica* (70%) (Fig. 1b), which corresponds to “LADH” purified from yeast cells grown on l-arabinose as a sole carbon source^40^. In this regard, PsXDH is suitable for generating a bifunctional dehydrogenase for xylitol and l-arabinitol, based on the structural data in this study, which is useful for breeding d-xylose and l-arabinose co-fermenting *S. cerevisiae*.

## Materials and methods

### Expression and purification of recombinant proteins

The primer sequences used in the present study are shown in Supplementary Table 2. Each (putative) XDH gene of *P. stipitis* (encoded by PICST_86924 gene), *S. cerevisiae* (YLR070c), and *S. pombe* (SPBC1773.05c) was introduced into pQE-81L (Qiagen), a plasmid vector for conferring an N-terminal (His)_6_-tag on expressed proteins, to yield pQE-PsXDH_WT_, pQE-ScXDH_WT_, and pQE-SpXDH_WT_, respectively. *E. coli* strain DH5α harboring the pQE-based vector was grown at 37 °C to a turbidity of 0.8 at 600 nm in LB medium containing ampicillin (50 mg/l). After the addition of 1 mM isopropyl-β-d-thiogalactopyranoside, the culture was grown at 37 °C for 6 h or at 20 °C for 18 h to induce the expression of the respective (His)_6_-tagged protein. Cells were harvested and resuspended in Buffer A (50 mM sodium phosphate buffer (pH 8.0) containing 300 mM NaCl and 10 mM imidazole). Cells were then disrupted by sonication and the solution was centrifuged. The supernatant was loaded onto a Ni–NTA Superflow column (Qiagen), which was then washed with Buffer B (pH 8.0, Buffer A containing 25 mM imidazole instead of 10 mM imidazole). Enzymes were eluted with Buffer C (pH 8.0, Buffer A containing 250 mM imidazole instead of 10 mM imidazole), and the elutant was loaded onto a HiLoad 16/600 Superdex 200 pg column (GE Healthcare) equilibrated with Buffer D (20 mM Tris–HCl (pH 8.0) containing 150 mM NaCl). The main single-peak fractions were collected and concentrated by ultrafiltration with Amicon Ultra-15 (Millipore).

### Site-directed mutagenesis

Several mutants of PsXDH, ScXDH, and SpXDH were constructed by a PCR-based method with the mutated sense and antisense primers (Supplementary Table S2), and pQE/PsXDH_WT_, pQE/ScXDH_WT_, or pQE/SpXDH_WT_ as a template, respectively.

### Enzyme assay

Dehydrogenase activity for xylitol was measured using a continuous spectrophotometric assay at 340 nm at 30 °C in 50 mM Tris–HCl buffer (pH 8.0) containing 100 mM xylitol and 1 mM NAD^+^.

### Crystallization and X-ray crystallography

All crystallization trials were performed at 20 °C using the sitting-drop vapor diffusion method. Drops (0.5 μL) of ~ 20 mg/mL PsXDH_C4_ protein in Buffer D were mixed with equal amounts of reservoir solution, and equilibrated against 70 μL of the same reservoir solution by vapor diffusion. The initial trial was performed using Index HT and Crystal Screen (Hampton Research). The best crystal of PsXDH_C4_ was obtained within 1 week under the following conditions: 100 mM Hepes–NaOH (pH 7.0), 2 M ammonium sulfate, and 2.5% (w/v) polyethylene glycol 400. The crystals obtained were cryoprotected with reservoir solution supplemented with 15% (w/v) glycerol, and flash-cooled and kept in a stream of nitrogen gas at 100 K during data collection.

Diffraction data were collected with the PILATUS 6 M detector of BL45XU at SPring-8 (Hyogo, Japan), and the processed ZOO system and XDS^41–43^. The structure of the apo-form of PsXDH_C4_ was solved by the molecular replacement method using the molecular-replacement pipeline program BALBES^44^ with the structure of SDH from sheep liver (PDB ID 3QE3)^11^ as the search model. Further model building for all structures was performed manually with COOT^45^ and crystallographic refinement with PHENIX^46^. Detailed data collection and processing statistics are shown in Table 1.

### Overexpressing XDH genes in *S. cerevisiae*

Each DNA fragment of (His)_6_-PsXDH_WT_, (His)_6_-PsXDH_C4_, (His)_6_-SpXDH_WT_, and (His)_6_-SpXDH_C100S_ was amplified by PCR using the pQE-based vector as a template and was then introduced into EcoRI-HindIII sites between the PGK expression cassettes in the plasmid YEpPGK^37^. *S. cerevisiae* D452-2 strains (*MATa leu2 his3 ura3 can1*) harboring the YEpPGK-based vector were grown in minimal medium supplemented with 2% (w/v) glucose as a sole carbon source at 30 °C. Cells were harvested, resuspended in 50 mM Tris–HCl (pH 8.0), and vortexed together with an equal volume of glass beads (diameter of 0.5 mm). Cell debris and glass beads from the cell extract were separated by centrifugation and the remaining supernatant was used for enzyme assessments.

### Sequence comparison

Protein sequences were analyzed using the Protein-BLAST and Clustal W programs distributed by the Kyoto Encyclopedia of Genes and Genomes (KEGG) of Japan (www.kegg.jp/kegg/kegg1.html)^47,48^.

## Supplementary Information

Supplementary Information 1.

Supplementary Information 2.

Supplementary Information 3.

Supplementary Information 4.

## Data Availability

Coordinates and structural factors have been deposited in the Protein Data Bank (www.pdb.org), under the accession code 7Y9P.
