# Supplementary material for: Molecular evolutionary insight of structural zinc atom in yeast xylitol dehydrogenases and its application in bioethanol production by lignocellulosic biomass
Source: Sci Rep. 2023 Feb 2;13:1920. doi: 10.1038/s41598-023-29195-7 (PMC9895041; doi:10.1038/s41598-023-29195-7)
Supplement: Supplementary file 1 — Supplementary Information 1. [file 41598_2023_29195_MOESM1_ESM.pdf]

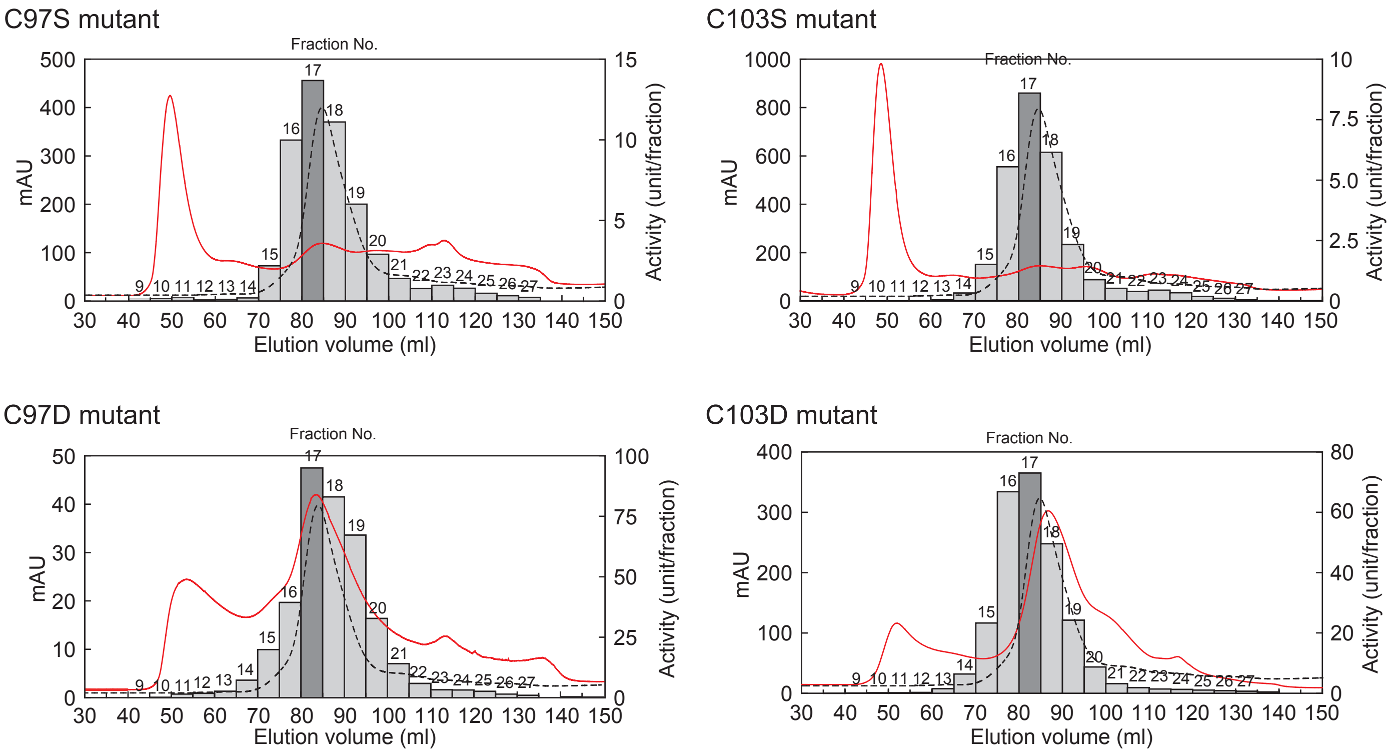

Figure S1. Elution profile of gel-filtration of PsXDH. Absorbance at 280 nm of each mutant and WT are shown as red and dash black lines, respectively. Bar graph indicates enzyme activity in each fraction (5 ml).
