# Supplementary figures and images for: Molecular evolutionary insight of structural zinc atom in yeast xylitol dehydrogenases and its application in bioethanol production by lignocellulosic biomass
Source: Sci Rep. 2023 Feb 2;13:1920. doi: 10.1038/s41598-023-29195-7 (PMC9895041; doi:10.1038/s41598-023-29195-7)

— Membrane edge

a

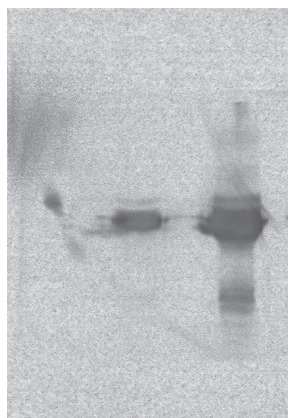

b

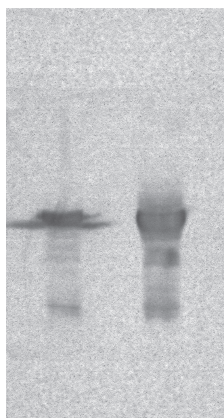

c

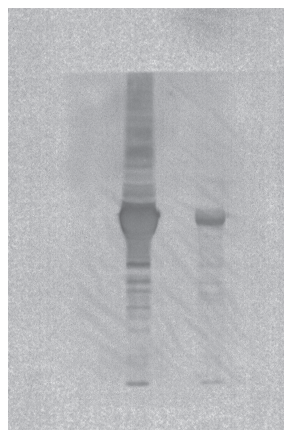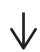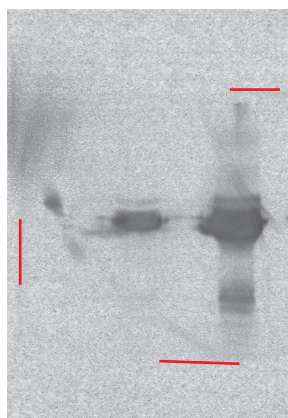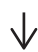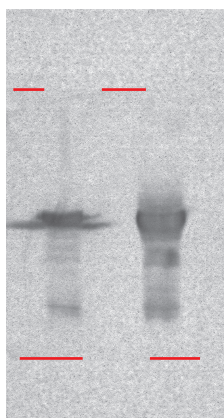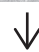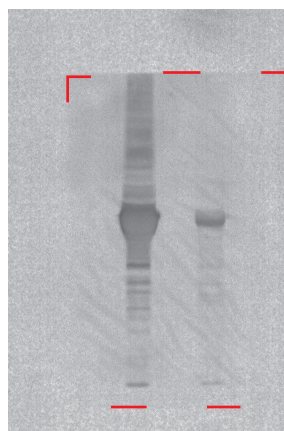

d

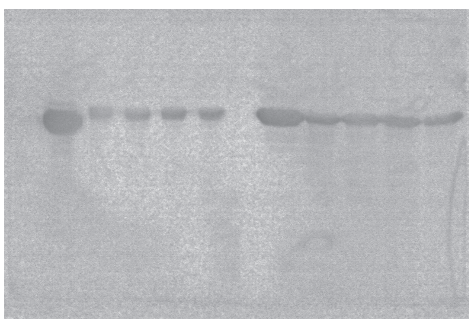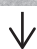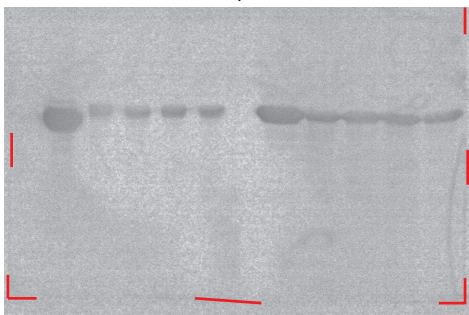

e

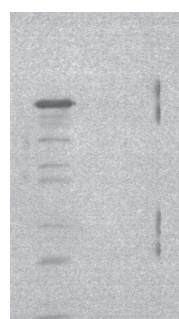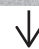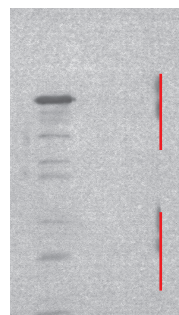

Supplement: Supplementary file 2 — Supplementary Information 2. [file 41598_2023_29195_MOESM2_ESM.pdf]
