# Supplementary material for: Molecular evolutionary insight of structural zinc atom in yeast xylitol dehydrogenases and its application in bioethanol production by lignocellulosic biomass
Source: Sci Rep. 2023 Feb 2;13:1920. doi: 10.1038/s41598-023-29195-7 (PMC9895041; doi:10.1038/s41598-023-29195-7)
Supplement: Supplementary file 3 — Supplementary Information 3. [file 41598_2023_29195_MOESM3_ESM.pdf]

Table S1. Comparison of crystal structures of PsXDH with other MDR enzymes.

Enzyme		Source organisms	Accession No.	r.m.s.d. (Å)	Number of C_α	Identity (%)	Tertiary structure	Structural zinc
PDH	XDH	*Pichia stipites*	7Y9P	-	-	-	4	No
	SDH	*Bemisia argentifolii*	1E3J	1.3	339	42	4	Yes
	SDH	Human	1PL7	1.6	341	41	4	No
	SDH	Sheep liver	3QE3	1.6	341	41	4	No
	LADH	*Neurospora crassa*	3M6I	2.2	343	36	4	Yes
TDH		*Pyrococcus horikoshii*	2DFV	2.4	341	31	4	Yes
ADH		Horse liver	8ADH	2.8	334	27	2	Yes
		Human	1HT0	3.5	335	27	2	Yes
		Arabidopsis thaliana	4RQT	3.3	339	25	2	Yes
		Tomato	4DL9	3.3	335	27	2	Yes
		Saccharomyces cerevisiae	4W6Z	2.7	347	23	4	Yes
		Pichia pastoris	5YAT	2.3	335	23	2	No
		Bacillus stearothermophilus	6IQD	2.1	331	26	2	Yes
		Ralstonia eutropha	3S1L	2.1	334	23	4	Yes
		Pseudomonas aeruginosa	1LLU	2.3	333	25	4	Yes
		Moraxella sp.	4Z6K	2.1	332	24	4	Yes
		Lactococcus lactis	4EEX	2.5	331	26	2	Yes
		Escherichia coli	4GKV	2.6	329	25	4	Yes
		Sulfolobus solfataricus	1JVB	2.3	334	22	4	Yes
		Pyrobaculum aerophilum	4JBG	2.7	325	23	4	Yes
		Aeropyrum pernix	1H2B	3.1	334	21	4	Yes
		Thermoanaerobacter brockii	1YKF	2.7	337	25	4	No
		Clostridium beijerinckii	1KEV	3.0	336	25	4	No
		Arabidopsis thaliana	2CF5	2.3	335	20	2	Yes
		Thermus sp. ATN1	4CPD	2.4	338	29	2	Yes
		Streptococcus pneumoniae	5YLN	2.7	333	29	2	Yes
		Human	1TEH	3.2	338	25	2	Yes
		Saccharomyces cerevisiae	1Q1N	2.4	332	21	2	Yes
