# Supplementary material for: Molecular evolutionary insight of structural zinc atom in yeast xylitol dehydrogenases and its application in bioethanol production by lignocellulosic biomass
Source: Sci Rep. 2023 Feb 2;13:1920. doi: 10.1038/s41598-023-29195-7 (PMC9895041; doi:10.1038/s41598-023-29195-7)
Supplement: Supplementary file 4 — Supplementary Information 4. [file 41598_2023_29195_MOESM4_ESM.pdf]

Table S2. Primers used in the present study.

Primer	Sequence[*]
Cloning of the PsXDH gene into pQE-81L	
P1 (BamHI)	5′-catggatccgACTGCTAACCCTTCCTTGGTGTTG-3′
P2 (HindIII)	5′-attaagctTTACTCAGGGCCGTCAATGAGACAC-3′
Cloning of the ScXDH gene into pQE-81L	
P3 (BamHI)	5′-catggatccgACTGACTTAACTACACAAGAAGCTATT-3′
P4 (HindIII)	5′-attaagctTCATTCCGGGCCCTCAATGATCGTCTTG-3′
Cloning of the SpXDH gene into pQE-81L	
P5 (BamHI)	5′-catggatccgGCACCTGCAGAGAAAGCATTCGTCTTG-3′
P6 (HindIII)	5′-attaagcttCTACGCATCGGGACCACCAATGATTAC-3′
Site-directed mutagenesis in the PsXDH gene[§]	
P7 (S96C/S99C/Y102C)	Ref. 22
P8 (S96D/S99C/Y102C)	5′-CCAGGTATTCCAgaTAGATTCTGTG-3′
P9 (S96C/F98R/S99C/E101F/Y102C)	Ref. 24
Site-directed mutagenesis in the ScXDH gene[§]	
P10 (D99C/S102C/M105C/D113C)	5′-CTGGGCCTGCAGaGCGGTGGTTCGG-3′
Site-directed mutagenesis in the SpXDH gene[§]	
P11 (C97S)	5′-CCCGGTTGCGTTaGTCGTTTATGCG-3′
P12 (C100S)	5′-GTTTGTCGTTTAaGCGATTACTGTC-3′
P13 (C103S)	5′-GTTTATGCGATTACaGTCGTTCTGG-3′
P14 (C111S)	5′-GATACAACTTGaGTCCTCATATGG-3′
P15 (C97R)	5′-CCCGGTTGCGTTcGTCGTTTATGCG-3′
P16 (C97E)	5′-CCCGGTTGCGTTgaaCGTTTATGCG-3′
P17 (C97D)	5′-CCCGGTTGCGTTgaTCGTTTATGCG-3′
P18 (C100D)	5′-GTTTGTCGTTTAgatGATTACTGTC-3′
P19 (C103D)	5′-GTTTATGCGATTACgaTCGTTCTGG-3′
P20 (C111D)	5′-GATACAACTTGgaTCCTCATATGG-3′
Cloning of the $(His)_6$- tagged PsXDH gene into YEpPGK	
P21 (EcoRI)	5′-acagaattcATGAGAGGATCTCACCATCACC-3′
P22 (HindIII)	The same as P2.
Cloning of the $(His)_6$- tagged SpXDH gene into YEpPGK	
P23 (EcoRI)	The same as P21.
P24 (HindIII)	The same as P6.
[*]Lower case letters indicate additional bases for introducing the digestion sites of restriction enzymes in parentheses.	
[§]Only sense primers are shown. Underlining indicates mutated regions.	
